# Andreas F Widmer for the Basel infection control team

**DOI:** 10.1186/1753-6561-5-S6-P123

**Published:** 2011-06-29

**Authors:** AF Widmer, Infection control team

**Affiliations:** 1 Infectious disease and Hospital epidemiology, University of Basel, Basel, Switzerland

## Introduction / objectives

Hand hygiene belongs to the basic components of any infection control program. Compliance is still an important issue, but the hand hygiene technique has gathered little attention. Proper technique of hand hygiene significantly improves bacterial killing, but few studies addressed this issue.

## Methods

The University of Basel hospitals is a 900 bed tertiary care center with 5 Intensive CUs and kidney and bone marrow transplant program. After introduction of the alcoholic hand rub in 1970, hand washing has been replaced with the alcoholic hand rub in >90%. Since 2007, health care workers and medical students are routinely trained to apply the proper technique.

## Results

1’030 observations were made in different wards, emergency rooms, ICUs and transplant units. Overall compliance with all 6 steps was 13.4%. The steps focusing on the fingertips and the thumb were frequently missed, namely 83-90% for nurses and 95-97% for physicians (p<0.05).

## Conclusion

The compliance with hand hygiene technique requires more focus and training: the thumb and the fingertips are frequently not adequately in contact with alcohol. Several techniques have been proposed, but basic training should ensure that the fingertips and the thumb are not missed during the alcoholic rub-in.

## Disclosure of interest

A. Widmer Grant/Research support from Ecolab, Switzerland, Speaker's Bureau of 3m, Switzerland.

